# SAXS fingerprints of aldehyde dehydrogenase oligomers

**DOI:** 10.1016/j.dib.2015.10.017

**Published:** 2015-10-25

**Authors:** John J. Tanner

**Affiliations:** Departments of Biochemistry and Chemistry, University of Missouri-Columbia, Columbia, MO 65211, United States

**Keywords:** Small-angle X-ray scattering, X-ray crystallography, Aldehyde dehydrogenase, Protein oligomeric state

## Abstract

Enzymes of the aldehyde dehydrogenase (ALDH) superfamily catalyze the nicotinamide adenine dinucleotide-dependent oxidation of aldehydes to carboxylic acids. ALDHs are important in detoxification of aldehydes, amino acid metabolism, embryogenesis and development, neurotransmission, oxidative stress, and cancer. Mutations in genes encoding ALDHs cause metabolic disorders, including alcohol flush reaction (ALDH2), Sjögren–Larsson syndrome (ALDH3A2), hyperprolinemia type II (ALDH4A1), γ-hydroxybutyric aciduria (ALDH5A1), methylmalonic aciduria (ALDH6A1), pyridoxine dependent epilepsy (ALDH7A1), and hyperammonemia (ALDH18A1). We previously reported crystal structures and small-angle X-ray scattering (SAXS) analyses of ALDHs exhibiting dimeric, tetrameric, and hexameric oligomeric states (Luo et al., Biochemistry 54 (2015) 5513–5522; Luo et al., J. Mol. Biol. 425 (2013) 3106–3120). Herein I provide the SAXS curves, radii of gyration, and distance distribution functions for the three types of ALDH oligomer. The SAXS curves and associated analysis provide diagnostic fingerprints that allow rapid identification of the type of ALDH oligomer that is present in solution. The data sets provided here serve as a benchmark for characterizing oligomerization of ALDHs.

Specifications table [please fill in right-hand column of the table below]

**Table:** 

Subject area	*Chemistry, Biology*
More specific subject area	*Biochemistry, Structural Biology*
Type of data	*SAXS data files (.dat) and protein structure coordinate files (.pdb)*
How data was acquired	*Small-angle X-ray scattering (SAXS) data collected at Advanced Light Source Beamline 12.3.1*
Data format	*Buffer-subtracted, merged experimental scattering curves (.dat)*
Experimental factors	*Purified protein samples were subjected to size exclusion chromatography and shipped at 4* *°C in 96-well trays to beamline 12.3.1.*
Experimental features	*Data were collected by the beamline staff as part of the mail-in SAXS program at the SIBYLS beamline. The beamline staff provides the user with buffer-subtracted SAXS curves. The user then performs subsequent data analysis and interpretation.*
Data source location	*Lawrence Berkeley National Laboratory, Berkeley, CA, USA*
Data accessibility	*SAXS curves and coordinates of crystal structures are provided as supplementary content*

## 1 Value of the data [describe in 3–5 bulleted points why this data is of value to the scientific community]

• SAXS is a robust method for determining the oligomeric states of proteins in solution.
• When combined with crystal structures, SAXS can also be used to determine quaternary structure.
• The dimeric, tetrameric, and hexameric forms of ALDH have distinctive SAXS curves and SAXS-derived structural parameters.
• SAXS provides a diagnostic fingerprint of ALDH oligomeric state and quaternary structure.
• The data sets provided here serve as a benchmark for characterizing ALDH oligomerization.

## 2 Data, experimental design, materials and methods

### 2.1 Representative examples of ALDH oligomers

SAXS fingerprints are provided for prototypical dimeric, tetrameric, and hexameric ALDHs. No other oligomeric forms of ALDH have been described to date.

**Fig. 1:**
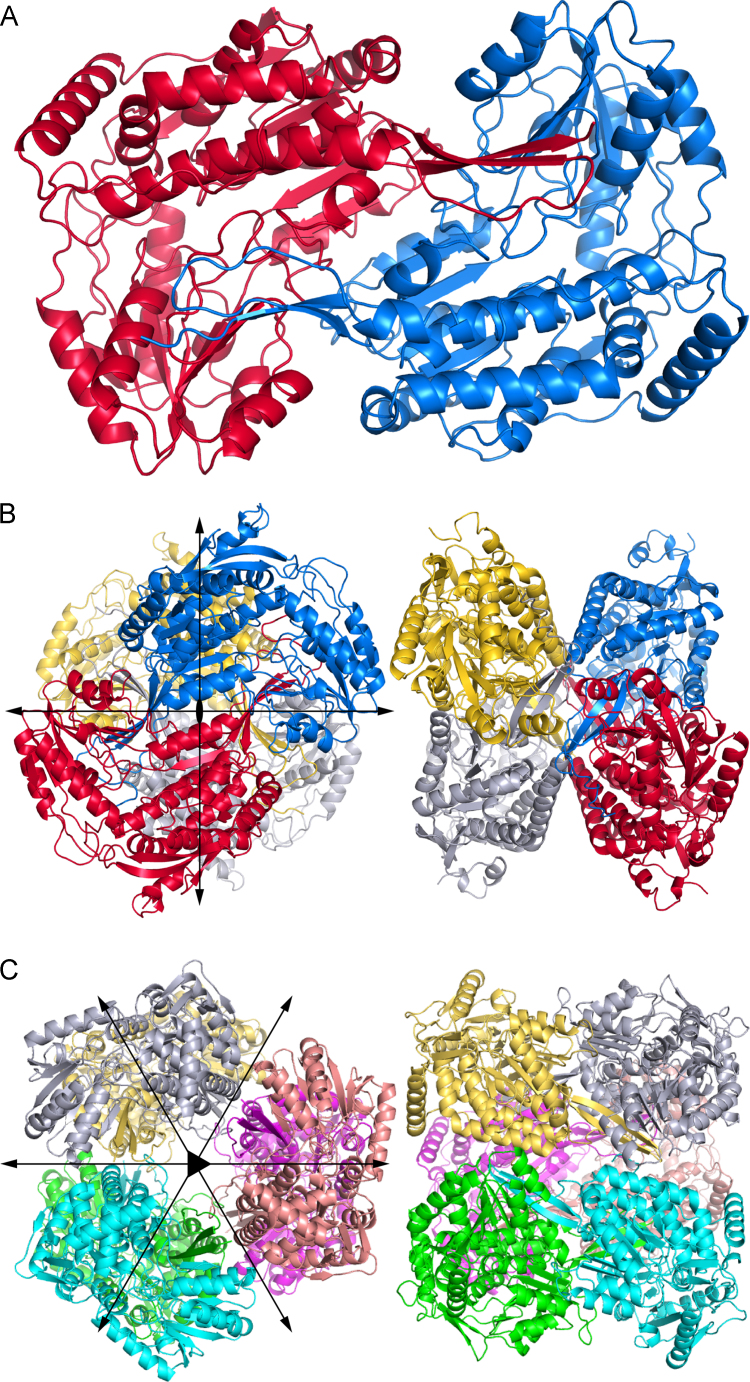
The three oligomers of the ALDH superfamily. (A) BhP5CDH is an example of a dimeric ALDH (PDB code 3QAN). (B) Human ALDH7A1 is a dimer-of-dimers tetramer (PDB code 4ZUL). Two orthogonal views are shown. The filled oval and arrows represent the three molecular 2-fold axes of the tetramer. (C) TtP5CDH forms a trimer-of-dimers hexamer (PDB code 2BHQ). Two orthogonal views are shown. The triangle represents the molecular three-fold axis, while the arrows represent the three molecular 2-fold axes. In all three panels, each chain has a different color.

*Bacillus halodurans* Δ^1^-pyrroline-5-carboxylate dehydrogenase (BhP5CDH) is presented here as an example of a dimeric ALDH. P5CDHs are part of proline catabolism and catalyze the oxidation of L-glutamate-γ-semialdehyde to L-glutamate [1]. P5CDHs belong to ALDH family 4 (member A1) and are also known as ALDH4A1. The ALDH dimer consists of two domain-swapped protomers and is the fundamental building block of higher order ALDH oligomers (Fig. 1A). The BhP5CDH dimer corresponds to chains A and B of the *C*2 asymmetric unit of PDB entry 3QAN; the coordinates of this dimer are provided in Supplementary material. Other examples of dimeric ALDHs include human and mouse ALDH4A1 [2], [3].

Human ALDH7A1 (hALDH7A1) forms the classic ALDH tetramer. ALDH7A1 is part of lysine catabolism and is also known as α-aminoadipate semialdehyde dehydrogenase [4], [5], [6]. The tetramer is a dimer of dimers having 222 symmetry (Fig. 1B). The crystal structure of hALDH7A1 complexed with α-aminoadipate (PDB entry 4ZUL [5]) has two equivalent tetramers in the *C*2 asymmetric unit; the coordinates of one of these tetramers are provided in Supplementary material. Other examples of tetrameric ALDHs include ALDH1 and ALDH2 [7].

*Thermus thermophilus* Δ^1^-pyrroline-5-carboxylate dehydrogenase (TtP5CDH) represents hexameric ALDHs [8]. The hexamer is a trimer of dimers (Fig. 1C). The crystal structure of TtP5CDH (PDB entry 2BHQ [9]) has a dimer in the *H*3 asymmetric unit. Application of the crystallographic 3-fold rotation generates the hexamer; the coordinates of this hexamer are provided in Supplementary material. Other hexameric ALDHs include the P5CDHs from yeast [10] and *Deinococcus radiodurans* [8].

### 2.2 Preparation of protein samples for SAXS data collection

Expression and purification of BhP5CDH, hALDH7A1, and *Thermus thermophilus* P5CDH (TtP5CDH) were described previously [5], [8]. Prior to SAXS analysis, each protein was passed through a Superdex 200 size exclusion column to remove any aggregated protein. We note that aggregation must be avoided as it greatly diminishes SAXS data quality, and in some cases, can cause the data to be unusable [11], [12]. Effluent from the size exclusion column was reserved for measurement of the background scattering. The protein samples and corresponding buffer samples were pipetted into 96-well PCR plates (Corning Axygen, VWR catalog number 10011-228). Each protein sample was included at three nominal concentrations in the range of 1–10 mg/mL. The total volume in each well was 30 μL. The trays were sealed with a silicone lid (Corning Axygen, VWR catalog number 10011-130). Each sealed tray was sandwiched between two cold packs that had been incubated at 4 °C, and the assembly was stabilized with rubber bands. The assembly was then placed in a Styrofoam box containing additional cold packs (at 4 °C) and sent via overnight express courier to beamline 12.3.1 of the Advanced Light Source.

### 2.3 SAXS data collection and analysis

SAXS data were collected by the beamline staff through the SIBYLS beamline mail-in program (bl1231.als.lbl.gov/htsaxs) [13], [14]. For each protein concentration, exposure times of 0.5, 1.0, 3.0, and 6.0 s were used. Scattering curves collected from the protein samples were corrected for background scattering using intensity data collected from the SEC effluent. Composite scattering curves for each protein concentration were generated with PRIMUS [15] by scaling and merging the background-corrected high *q* region from the 3 s exposure with the low q region from the 0.5 s or 1.0 s exposure. PRIMUS was also used for Guinier analysis. GNOM was used to calculate distance distribution functions [16]. Composite scattering curves for BhP5CDH, ALDH7A1, and TtP5CDH are provided in Appendix A, Appendix A.

### 2.4 SAXS fingerprints of ALDH oligomers

**Fig. 2:**
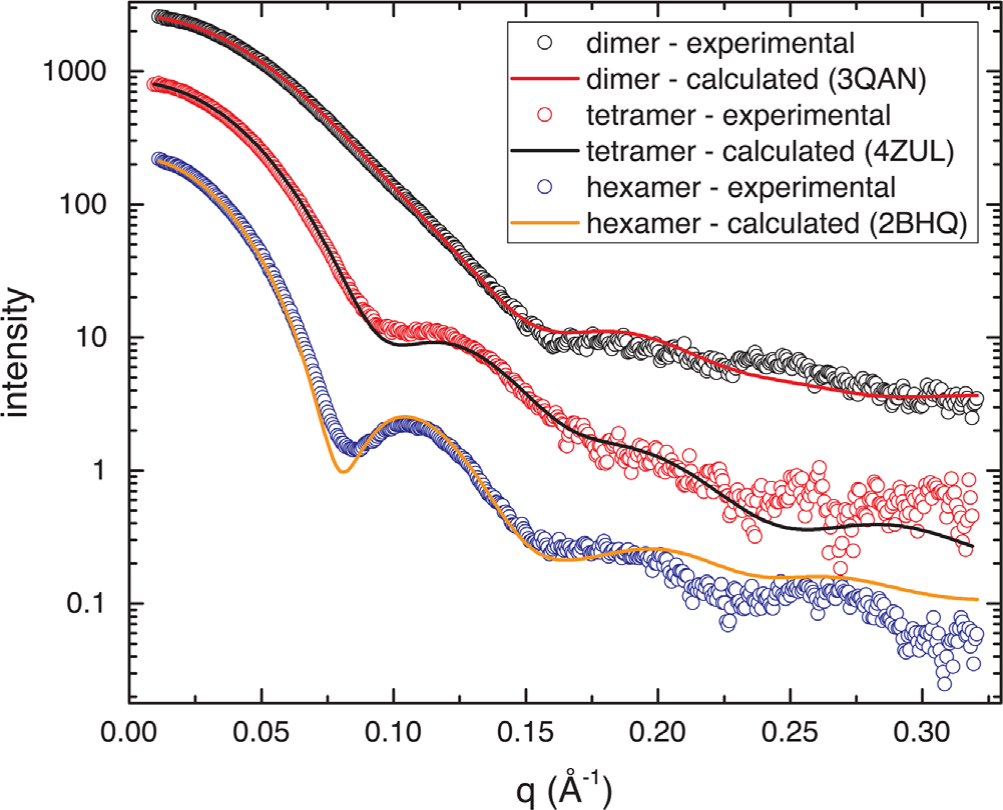
SAXS data for dimeric (BhP5CDH, black circles), tetrameric (hALDH7A1, red circles), and hexameric (TtP5CDH, blue circles) ALDHs. The smooth curves are theoretical SAXS data calculated from the atomic models provided in the Supplement using FoXS [21]. The fit quality parameters (*χ*) are 2.2 for the dimer, 1.5 for the tetramer, and 5.5 for the hexamer. Arbitrary scale factors have been applied so that the data sets are offset for ease of comparison.

SAXS curves for BhP5CDH (dimer), hALDH7A1 (tetramer), and TtP5CDH (hexamer) are shown in Fig. 2. The dimer curve is distinct from the others in that it is relatively featureless and monotonically decreasing with *q* in the region of *q*<0.15 Å^−1^. In contrast, the tetramer and hexamer curves show peak and valley features in the region *q*=0.075–0.15 Å^−1^. These features are more pronounced in the hexamer curve.

The radius of gyration (*R*_g_) is a fundamental solution structural parameter that is quickly determined from SAXS data. *R*_g_ can be estimated from Guinier analysis or calculation of the distance distribution function [12], [17]. Using either method, one finds that *R*_g_ increases with increasing degree of oligomerization (Table 1). The Guinier *R*_g_ values estimated with Primus using the supplied data files are 31.2±0.1 Å for the dimer (using *qR*_g_ range of 0.35–1.30), 37.9±0.5 Å for the tetramer (*qR*_g_=0.36–1.30), and 43.4±0.3 Å for the tetramer (q*R*_g_=0.49–1.28). The *R*_g_ values from calculations of the distance distribution function (real space *R*_g_) are in good agreement with those from Guinier analysis (Table 1). Furthermore, the SAXS *R*_g_ values agree well with those calculated from the crystal structures (Table 1).

The molecular mass of proteins in solution can be calculated from SAXS data using the volume of correlation method of Rambo and Tainer [18]. The molecular masses calculated from the ALDH data sets are in good agreement with the theoretical values (Table 1).

Theoretical SAXS data can be calculated from atomic coordinates obtained from crystal structures or homology models. Comparison of the experimental and calculated data allows determination of the quaternary structure of the oligomer in solution. The theoretical SAXS data calculated from the supplied oligomer crystal structure models agree well with the experimental SAXS data (Fig. 2).

**Fig. 3:**
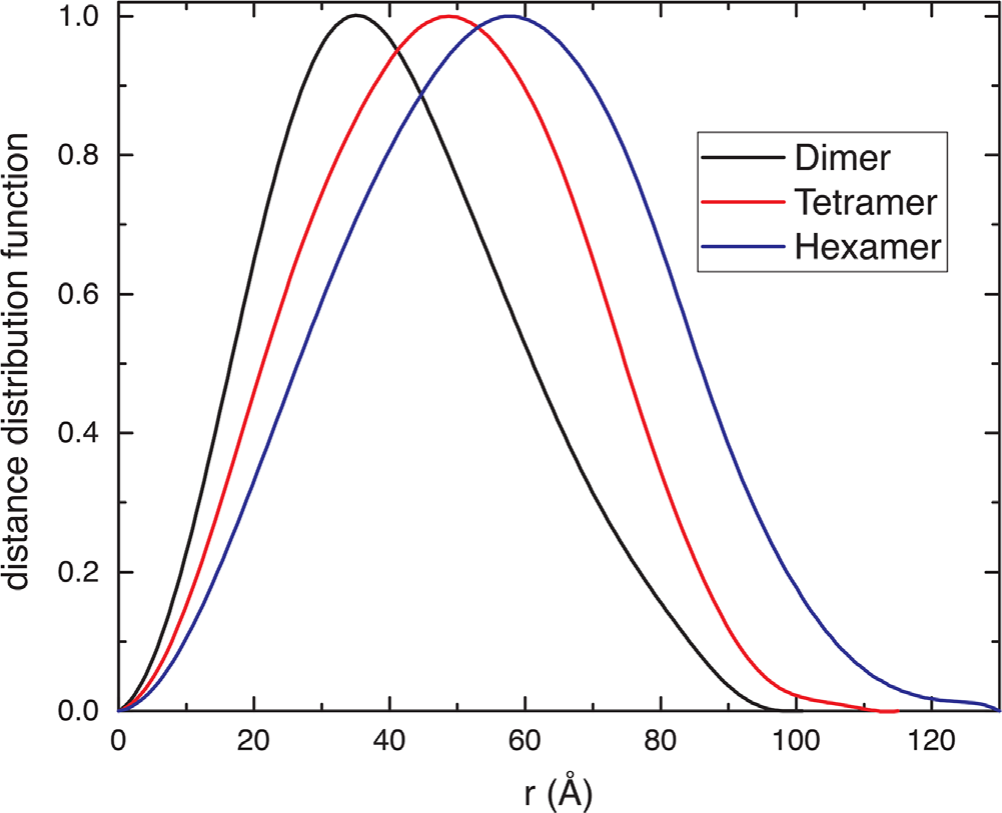
Distance distribution functions for dimeric (BhP5CDH, black), tetrameric (hALDH7A1, red), and hexameric (TtP5CDH, blue) ALDHs.

The distance distribution function is another aspect of the SAXS fingerprints of ALDH oligomers (Fig. 3). In each case, the distribution function exhibits a single maximum; however, the oligomers can be distinguished by the position of the maximum, the width of the distribution, and the maximum particle dimension (*D*_max_). The position of the maximum increases with increasing degree of oligomerization, from *r*=36 Å for the dimer, to *r*=49 Å for the tetramer, and *r*=58 Å for the hexamer. Also, the distribution widens with increasing degree of oligomerization. The peak width at half-maximum is 45 Å for the dimer, 53 Å for the tetramer, and 58 Å for the hexamer. *D*_max_ is the distance at which the distribution function decays to zero. This value is smallest for the dimer (95–105 Å), intermediate for the tetramer (105–120 Å), and largest for the hexamer (120–125 Å).

In summary, the three oligomeric forms of ALDH are readily distinguishable from SAXS. The data supplied here serves as a benchmark for characterizing ALDH oligomerization.

## Figures and Tables

**Table 1:** Radii of gyration of ALDH oligomers.

Representative ALDH	Oligomeric state	Guinier *R*_G_ (Å)	Real space *R*_G_ (Å)	Crystal structure *R*_G_ (Å)^a^	*M* (kDa)^b^
BhP5CDH	Dimer	31	32	31	91 (115)
hALDH7A1	Tetramer	38	37	36	175 (222)
TtP5CDH	Hexamer	43	43	43	309 (343)

a Calculated from the coordinates provided in Appendix A, Appendix A using MOLEMAN [19].

b Molecular mass of the oligomer in solution calculated from the SAXS data using the volume of correlation method as implemented in Scatter 1.0 [20]. The theoretical mass calculated from the amino acid sequence is listed in parentheses.

## Appendix A Supplementary material

SAXS curves for BhP5CDH, TtP5CDH, and ALDH7A1 are provided as text files with file extension .dat. Coordinates of the BhP5CDH dimer, hALDH7A1 tetramer, and TtP5CDH hexamer are provided as text files with file extension.pdb.

## Footnotes

Appendix A Supplementary data associated with this article can be found in the online version at 10.1016/j.dib.2015.10.017.
